# AM fungal-bacterial relationships: what can they tell us about ecosystem sustainability and soil functioning?

**DOI:** 10.3389/ffunb.2023.1141963

**Published:** 2023-08-01

**Authors:** Shabana Hoosein, Lena Neuenkamp, Pankaj Trivedi, Mark W. Paschke

**Affiliations:** ^1^ Department of Forest and Rangeland Stewardship/Graduate Degree Program in Ecology, Colorado State University, Fort Collins, CO, United States; ^2^ Institute of Landscape Ecology, Münster University, Münster, Germany; ^3^ Department of Ecology and Multidisciplinary Institute for Environment Studies “Ramon Margalef,” University of Alicante, Alicante, Spain; ^4^ Microbiome Network, Department of Agricultural Biology, Graduate Degree Program in Ecology, Colorado State University, Fort Collins, CO, United States

**Keywords:** arbuscular mycorrhizal fungi, AM fungal-bacterial relationships, interactions, symbioses, microbial functional traits, biogeochemical cycling, synthetic communities

## Abstract

Considering our growing population and our continuous degradation of soil environments, understanding the fundamental ecology of soil biota and plant microbiomes will be imperative to sustaining soil systems. Arbuscular mycorrhizal (AM) fungi extend their hyphae beyond plant root zones, creating microhabitats with bacterial symbionts for nutrient acquisition through a tripartite symbiotic relationship along with plants. Nonetheless, it is unclear what drives these AM fungal-bacterial relationships and how AM fungal functional traits contribute to these relationships. By delving into the literature, we look at the drivers and complexity behind AM fungal-bacterial relationships, describe the shift needed in AM fungal research towards the inclusion of interdisciplinary tools, and discuss the utilization of bacterial datasets to provide contextual evidence behind these complex relationships, bringing insights and new hypotheses to AM fungal functional traits. From this synthesis, we gather that interdependent microbial relationships are at the foundation of understanding microbiome functionality and deciphering microbial functional traits. We suggest using pattern-based inference tools along with machine learning to elucidate AM fungal-bacterial relationship trends, along with the utilization of synthetic communities, functional gene analyses, and metabolomics to understand how AM fungal and bacterial communities facilitate communication for the survival of host plant communities. These suggestions could result in improving microbial inocula and products, as well as a better understanding of complex relationships in terrestrial ecosystems that contribute to plant-soil feedbacks.

## 1 Introduction

Land management practices have had a devastating toll on the health and sustainability of our soil systems, developing into the next big scientific hurdle of the century: how to mitigate soil degradation. (Keesstra et al., 2018; Eswaran et al., 2019; Bardgett et al., 2021). Loss of belowground biodiversity by land use change has a significant impact on soil functions and plant productivity (Wagg et al., 2014; Geisen et al., 2019; Kopittke et al., 2019; Hasan et al., 2020). Over the past decade, studies have indicated that symbiotic soil organisms (like arbuscular mycorrhizal fungi, or AM fungi) can alter the effects of land management (i.e., soil disturbance) by enhancing host plant survival, ecosystem services, and plant resilience (Caravaca et al., 2005; Martínez-García et al., 2017; Neuenkamp et al., 2019). For example, AM fungal associations with plants improves nutrient translocation, water retention, and prime plants against pathogens (Borowicz, 2001; Manoharan et al., 2010; Diagne et al., 2020). As an important player in the rhizosphere, AM fungi impact microbial assembly within the rhizosphere (Yuan et al., 2021), where its interactions with soil microbes influence ecosystem processes (Wang et al., 2022), soil formation (Herman et al., 2012; Anthony et al., 2020), and crop production (Artursson et al., 2006; Giovannini et al., 2020). Yet, research on the relationships between AM fungi and bacteria faces a major knowledge gap in understanding how soil microbial interactions independently and collectively contribute to soil function. A synthesis of the recent literature is needed to elucidate the different ways that AM fungal-bacterial relationships influence soil systems and how their collective traits contribute to the sustainability of soil function.

To effectively harness the microbiome, we need new approaches which recognize that those microbes living in natural and managed systems typically do so as communities - not populations of single organisms functioning alone (Trivedi et al., 2020). There is strong evidence that interactions of different organisms alter their function while also enhancing the persistence and resilience of microbial communities (Azcón-Aguilar and Barea, 1992; Fitter and Garbaye, 1994; Griffiths and Philippot, 2013; Felipe-Lucia et al., 2020). One can consider the entire functional entity of the plant and its associated microbiota as the *holobiont* (*see **Box 1** for term glossary*) (Vandenkoornhuyse et al., 2015; Singh et al., 2020). Hence, the plant holobiont is shaped by the tripartite symbiosis between plants, AM fungi, and bacteria (Garbaye, 1994; Artursson et al., 2006; Miransari, 2011; Jansa et al., 2013) revealing the complexity of these inter-dependent mutualisms in order to understand AM fungal community assembly, habitat filtering, and niche partitioning — i.e. their functioning.

AM fungi exert influence on soil microbial assembly by providing microhabitats and thereby sustain greater soil microbial diversity (Azcón-Aguilar and Barea, 1992; Fitter and Garbaye, 1994; Nazir et al., 2009; Miransari, 2011; Bach et al., 2018; Faghihinia et al., 2022). This increase in AM fungal-mediated microhabitats, in turn, provides habitats for functionally diverse soil microbes, faster litter decomposition and nutrient cycling (Herman et al., 2012; Nuccio et al., 2013), influencing biogeochemical cycling and ecosystem processes (van Elsas et al., 2006; Wagg et al., 2019; Sokol et al., 2022). The interaction between AM fungi and soil microbial communities, however, runs in both directions as soil microbial communities influence AM fungal development and growth, too (Azcón-Aguilar and Barea, 1992; Fitter and Garbaye, 1994). A third player joins this bidirectional Interaction between AM fungi and soil microbes, the host plants of AM fungi, through their provisioning of carbon (Artursson et al., 2006; Smith and Smith, 2011). Growing evidence across the literature shows that AM fungi have a direct effect on plant fitness (Smith and Smith, 2011) and abiotic attributes within the soil environment (Johnson et al., 1997; Jansa et al., 2013; Powell and Rillig, 2018), but research is only beginning to understand AM fungal interactions with soil microbes and its impact on soil functioning.

We can begin to process patterns and larger impacts of AM fungal-bacterial relationships by identifying mechanistic links between bacterial and AM fungal communities. The first studies suggested potential mechanisms for specific aspects of the interaction based on experimental results, e.g., that other soil microorganisms enhance AM fungal growth by removing their self-inhibitors (Watrud et al., 1978). Another recent way to identify mechanisms is by incorporating the drivers of microbial coexistence and through distinguishing persistence of each microbial group through microbial functional traits (Chaudhary et al., 2020). Some AM fungal functional traits (e.g., carbon acquisition from host plant or resilience of hyphal networks) have been hypothesized and categorized (van der Heijden and Scheublin, 2007, Chagnon et al., 2013; Chagnon et al., 2015; Chaudhary et al., 2020), but many of AM fungi’s taxonomic attributes (e.g., hyphal decomposition rate or spore production) have yet to be identified as functional traits within each AM fungal family. These gaps of knowledge, altogether, lead to more questions about the persistence and ecosystem effects of AM fungal-bacterial interactions in the soil, with the potential for larger implications in the microbial inoculum market and our fundamental knowledge of terrestrial ecosystem functioning. By uncovering the mechanisms regulating these interactions, we can begin to determine what conditions cause them to shift and how the compositional complexity within these distinct microbiomes relate to AM functional traits that support (positive or negative) *microbial interactions (see **Box 1**)*.

Box 1 **Glossary of Terms**

**Hyphosphere**: soil zone surrounding hyphae where biological, physical, and chemical activities are influenced by fungal hyphae**Holobiont**: the assemblage of organisms that occupy the space in and around the host, influencing host fitness and survival through interdependent and complex dynamics **(microbial) interactions**: patterns that contribute to microbes occupying the same niche space**(microbial) functionality**: attributes of a specific taxa or microbial community that contributes to holobiont survival and ecosystem processes**Functional diversity**: roles or attributes that taxa contribute to community function that sustain diverse biological processes and help withstand stressors **Metabolic plasticity**: the evolutionary adaptation of a microbial community to withstand environmental variations through the mediation of metabolites produced by surrounding organisms

In this review, we explore AM fungal relationships with bacterial communities within the rhizosphere microbiome, highlighting the importance of functional traits across AM fungal families which are imperative in understanding the long-term functioning and sustainability of managed soil systems. We also provide insights into pattern-based analyses, like network analyses, which have been effective in communicating data trends throughout other disciplines in recent years. We intend for this paper to highlight interdisciplinary research and cross-disciplinary collaborations that can help push AM fungal research towards solutions for the improvement and sustainability of degraded soil systems. We review current research on soil microbiomes, revealing the complexity behind AM fungal-bacterial interactions, their affiliation with their plant hosts, and why microbe-microbe relationships are important for plant health. Based on our literature synthesis, we provide suggestions for further studies that incorporate cooccurrence data to understand the ecology of AM fungal-bacterial relationships and identify a need for building empirical data to evaluate functional traits and their ecosystem consequences. We conclude that the community-oriented nature of soil microbiomes can bring awareness to the microbial relationships and how *microbial functionality (see **Box 1**)* is sustained in soil systems, through the incorporation of technologies across multiple disciplines.

## 2 Drivers and relevance of AM fungal-bacterial interactions in the rhizosphere

AM fungi interact with most organisms present in the rhizosphere, thereby connecting different soil microbiota and maintaining the functioning of soil systems (Jansa et al., 2013; Sokol et al., 2022). AM fungi communicate, signal, and interpret external stressors to the plant, which are then signaled by the plant to the soil microbial community (Miransari, 2011; Zhang et al., 2018a; Cornell et al., 2022). In this process AM fungi assist in this plant-bacterial signaling through molecular communications within the rhizosphere, priming bacterial communities for functions needed by plants.

### 2.1 AM fungal and bacterial cooperation in the rhizosphere

It is well known that AM fungi coevolved with plants over 400 million years ago (Bonfante and Genre, 2008; van der Heijden et al., 2015), but it is also hypothesized that AM fungal-bacterial relationships coevolved with the transition of plants to land (Garbaye, 1994; van Overbeek and Saikkonen, 2016; Olsson et al., 2017). At each trophic level of interacting organisms, microbial and symbiotic coevolutionary processes support the establishment and persistence of plant hosts and the success of plant-associated microbial communities. These coevolutionary processes between bacteria and AM fungi are evident in many forms within the rhizosphere including the production of antimicrobial/antifungal compounds, AM fungal fructose exudation, and host plant exudations that inhibit bacterial pathogens from dominating the rhizosphere microbiome (Bonfante and Genre, 2008; Willis and Rodrigues, 2013, Zhang et al., 2018b; Emmett et al., 2021). Bacteria and AM fungi are most abundant in the rhizosphere where metabolites are exuded by plants as a communicative bridge between and among soil microbes and plants (Boer et al., 2005; el Zahar Haichar et al., 2008). While bacteria and AM fungi occupy similar spatial niches, the distinct, yet the collaborative roles of bacterial-AM fungal relationships as an extension of the rhizosphere into the surrounding soil environment are worthy of greater attention (Vályi et al., 2016; Yuan et al., 2021).

Collaboration between bacteria and AM-fungi seems to be bilateral. Bacteria support the persistence of mycorrhiza through the inhibition of antagonistic fungal pathogens, promotion of AM fungal hyphal growth, and the protection of mycorrhizal associations by endophytic processes (van Overbeek and Saikkonen, 2016; Igiehon and Babalola, 2018). Some bacteria have been shown to increase AM fungal spore germination and symbiosis establishment with host plants (Giovannini et al., 2020). Isolates of actinobacteria (within the genera *Streptomyces* and *Corynebacterium*, amongst others) as well as mycorrhiza helper bacteria, have coevolved with AM fungi resulting in bacterial endophytes that decompose insoluble biopolymers that make up AM fungal spore walls, as well as enhancing AM fungal spore germination under the appropriate conditions (Bonfante and Genre, 2015; Martin et al., 2017; Turrini et al., 2018).

Due to their intimate association within plant roots, AM fungi have been known to influence the development of the soil microbial community (Chen et al., 2018; Zhang et al., 2018a). AM fungi benefit other soil microbes by encouraging growth of plant beneficial bacteria, such as plant-growth-promoting-rhizobacteria (PGPR) and mycorrhiza helper bacteria, which synergistically prevent antagonistic prokaryotic infections in the rhizosphere (Artursson et al., 2006; Frey-Klett et al., 2007; Scherlach et al., 2013). It is well established that AM fungi act as a major conduit of carbon transfer for soil bacterial communities (Drigo et al., 2010), influencing bacterial composition and structure (Zhang et al., 2018a). To access these nutritional hotspots, bacteria adhere to hyphal surfaces enabling them to spread throughout the soil environment (Hassani et al., 2018). These ‘fungal highways’ mobilize bacteria and thus increase their exposure to nutrients that are spread out in the bulk soil environment (Kohlmeier et al., 2005; Worrich et al., 2016; Jansa and Hodge, 2021; Jiang et al., 2021), enhancing plant acquisition of nutrients.

AM fungal-bacterial collaboration, however, is not a spontaneous act, but rather the result of long co-evolutionary processes. Bacteria’s coevolution with fungi is evident in bacteria’s resistance to antibacterial products produced by fungi, allowing bacteria to colonize near fungi (Boer et al., 2005), for example the nutritional hotspots exposed by AM fungal hyphae in the soil (Nazir et al., 2009; Wang et al., 2022). Researchers have found that mycorrhizal-associated bacteria inhibit fungal pathogens through the production of antibiotics or by secreting siderophores that outcompete pathogenic bacteria for iron (Garbaye, 1994; Turrini et al., 2018). There are also specific AM fungal characteristics that have coevolved with bacteria. One example is the surface of AM fungal hyphae that selects for those bacteria that excrete extracellular polymers to adhere to the hyphal surface (Bianciotto et al., 2001; Artursson et al., 2006). The extraradical mycelium of AM fungi hosts are a unique functional zone known as the *hyphosphere* (*see **Box 1***) (Zhang et al., 2022). Within the hyphosphere, AM fungal hyphae are known to release exudates that recruit microbes with various functions (Scheublin et al., 2010), creating an active zone of nutrient transformation (Kameoka et al., 2019; Zhang et al., 2022), where bacteria travel along fungal hyphae to reach these hotspots (Jiang et al., 2021; Jansa and Hodge, 2021). Extracellular polymeric substances in biofilms along hyphae prolong the survival rate and travel of bacterial cells and contribute to the vital components of microaggregate formation (Cai et al., 2019; Zethof et al., 2020). While more research is needed in these areas, understanding how AM fungal-bacterial relationships shift from facilitative to antagonistic can give insight into the sustainability of the ecosystem services this relationship provides.

### 2.2 The effect of molecular plant-AM fungal-bacterial communication in holobiont persistence

Besides supporting each other, recent research suggests that collaboration between AM fungi and bacteria contribute to increased survival and fitness of plants, which can be advantageous to host plants that struggle to adapt to changing environmental conditions (Bonfante and Anca, 2009; Bergmann et al., 2020). In utilizing *in vivo* and *in vitro* techniques, researchers have also found that co-inoculation of AM fungi and bacteria increases lead to the translocation of carbon from plants to bacteria *via* AM fungi (Kameoka et al., 2019; Emmett et al., 2021). Studies using PGPR have shown that the synergistic effect of co-inoculation with both AM fungi and *Pseudomonas* enhances host plant defenses (Pérez-de-Luque et al., 2017), increases host plant salinity tolerance (Moreira et al., 2020; Pan et al., 2020), and alleviates host plant stress from drought (Ghorchiani et al., 2018; Begum et al., 2019). In all cases co-inoculation was more effective than inoculation with either microbial group alone. Therefore, the interactions between fungi and bacteria provide more for the rhizosphere microbiome than each kingdom alone.

Signaling through the AM fungal hyphosphere confirms that plants influence metabolic exudation from the hyphosphere, thus changing the bacterial composition associated with the AM fungal hyphosphere (Cabral et al., 2019; Wang et al., 2022; Zhang et al., 2022). The mechanisms behind neighbor-induced triggers to increase plant defenses deserves more investigation. However, it seems that AM fungi are heavily involved in mediating communications from host plants to the soil environment (Cabral et al., 2019).

The pathways through which AM fungi “communicate” plant signals to the soil bacterial community are likely transcriptional changes that occur within mycorrhizal-associated plants (Miransari, 2011; Balestrini et al., 2017). These transcriptional changes lead to altered production of both primary and secondary metabolites i.e., the primary (nitrogen, protein, and carbohydrate pathways) and secondary metabolic pathways (root exudate pathway) (Sbrana et al., 2014; Zhang et al., 2018b; Zhang et al., 2022). Therefore, AM fungal interactions with host plants provide a pathway for the indirect regulation of bacterial communities in the hyphosphere.

More recent hypotheses suggest that AM fungi indirectly influence soil bacterial communities by influencing plant secondary metabolites as exuded by plant roots. While the effect of plant secondary metabolites on rhizosphere bacteria are often obscure, there have been several studies that have investigated the production of secondary metabolites in plants associated with AM fungi (Turrini et al., 2018; Szczałba et al., 2019). Associations with AM fungi change the amount of phenolic acid exudates released by plants, which contain antimicrobial properties (Pang et al., 2021; Wu et al., 2021). Moreover, Wu et al. (2021) found that specific AM fungal interactions, between two species (*Funneliformis geosporum* and *Acaulospora laevis*), reduced primary metabolic production in associated host plants, while all other combinations of mycorrhizal inoculum increased phenolic acid levels. Although AM fungal-induced changes in plant secondary metabolite production could indirectly decrease bacterial function, other AM fungal-induced increases in plant phenolic acid levels have been presumed to attract bacteria to the rhizosphere, imposing direct competition with the existing microbial community (Turrini et al., 2018; Giovannini et al., 2020; O’Banion et al., 2020).

Production of secondary metabolites due to plant associations with mycorrhizal fungi may also play a role in metabolic mutualism, or cross-feeding, amongst other microorganisms in the rhizosphere (Miransari, 2011; Zhang et al., 2018b; D’Souza et al., 2021). Rhizosphere bacteria have been known to synthesize their own secondary metabolites for microbial communications including anti-fungal, anti-bacterial, pigments that provide protection, and siderophores involved in scavenging iron (Turrini et al., 2018; Dror et al., 2020; Pang et al., 2021). While the role of AM fungi in these processes have yet to be elucidated, it is evident that AM fungi indirectly influence functions in host plants (like plant metabolite production) and have been thought to mimic quorum sensing in bacteria (Zhang et al., 2018b; Emmett et al., 2021; Duan et al., 2022). Nonetheless, AM fungi’s high biomass in most soil environments lend to ecological advantages that increase their interactions with organisms both within and outside the rhizosphere.

An additional factor that needs to be accounted for in AM fungal studies is the interactions between bacterial communities within the biological marketplace, as represented by a series of hyphal networks (Kiers et al., 2011; Fellbaum et al., 2012; Noë and Kiers, 2018), as hyphal networks provide a niche for bacterial establishment. For example, Bahram et al. (2020) found that soils dominated by AM fungi experience more nutrient turnover and cycling compared to ectomycorrhizal dominated soils suggesting that ecosystem function and plant benefits from AM fungal associations are reliant on the function of the entire holobiont and its associated microbiota.

### 2.3 AM fungal-bacterial relationships to plant and ecosystem functioning

**Figure 1:**
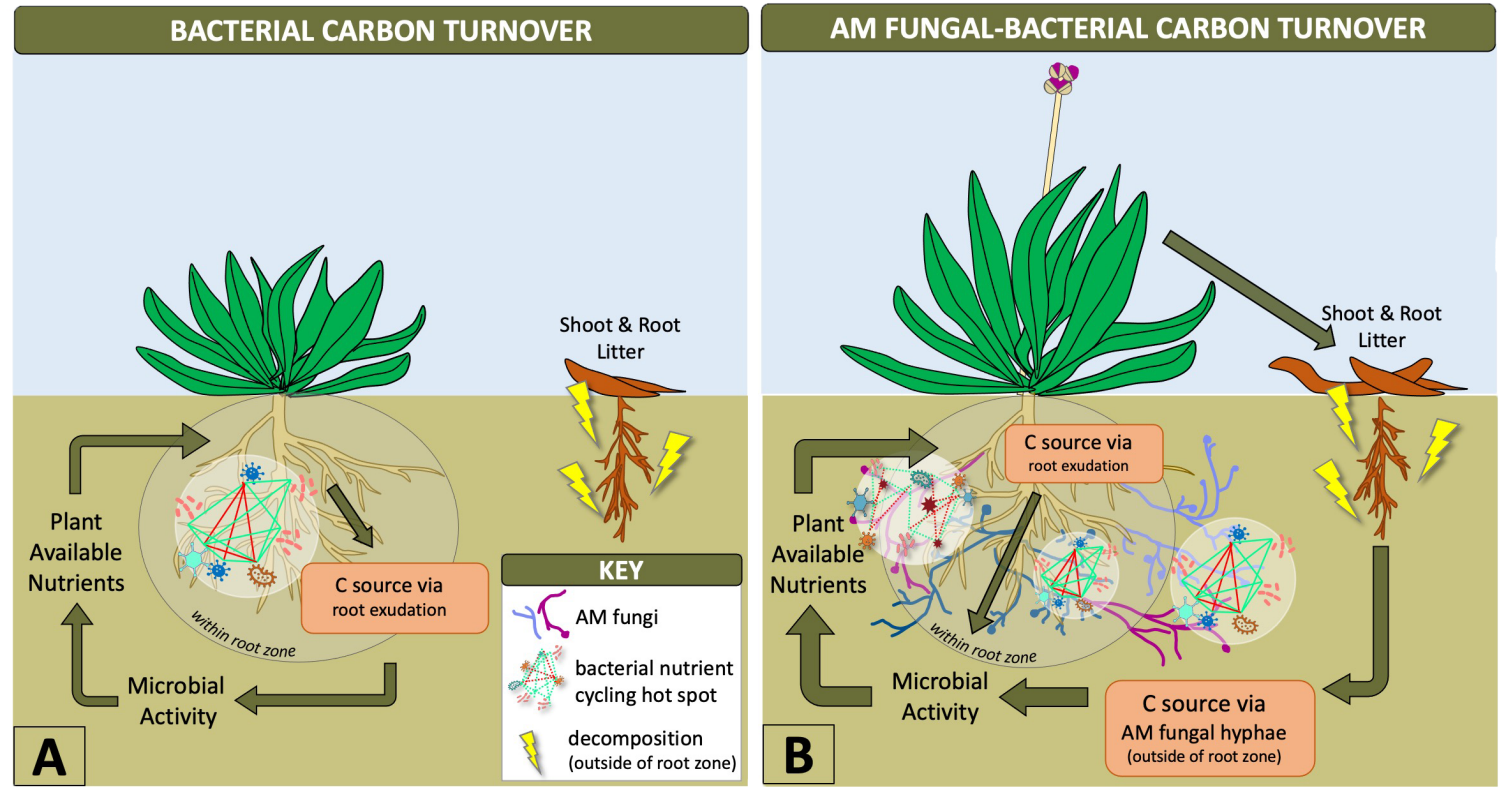
Diagram showing how AM fungal-bacterial relationships shift carbon turnover outside of the root zone where hyphal-associated bacteria can utilize more nutrients. Arrows with greater thickness indicate greater microbial contributions to plant-soil dynamics and aboveground-belowground cycling. AM fungal-bacterial relationships increase bacterial nutrient cycling hotspots beyond the rhizosphere due to carbon transportation from plants to AM fungal hyphae, which stimulate microbial activity and supply of diverse nutrients from outside of the root zone to plants **(B)**. Bacterial hotspots within the rhizosphere are limited by exuded carbon supply from roots, a metabolically intensive pathway for plants, which limits the supply of nutrients to those available within the root zone **(A)**.

Synergistic interactions between mycorrhizal fungi and bacteria help provide necessary nutrients for plant growth, such as phosphorus, which are mobilized by bacteria and taken up and transported to the host plant by AM fungal hyphae (Sharma et al., 2020; Wang et al., 2022). Outside of the rhizosphere, AM fungal hyphae provide the predominant source of plant carbon for soil microbes (Kaiser et al., 2015) stimulating a diversity of bacteria based on AM fungal genotype and hyphal exudation (Faghihinia et al., 2022), as depicted in **Figure 1**. This likely increases the number of nutritional hotspots outside of the rhizosphere by stimulating bacterial communities with labile C in an environment that contains mostly non-labile (recalcitrant) forms of carbon (Jansa et al., 2013; Faghihinia et al., 2022). The rapid turnover of mycorrhizal hyphae into soil C is known to be a fundamental source of plant-derived carbon transformation that increases the stability of soil organic matter (Godbold et al., 2006; Frey, 2019). AM fungal hyphae are also an important energy source, influencing carbon flux that drives hyphal-associated bacterial biochemical cycling within and outside of the root zone (Emmett et al., 2021; Wang et al., 2022) (**Figure 1**). Therefore, AM fungi have an indirect influence on biogeochemical cycling by creating niches outside of the rhizosphere (Yuan et al., 2021). This is of particular interest in agriculture where biochemical cycles related to nutrient availability to plants and plant productivity are the major objectives. Our current knowledge has been limited to *in vitro* experiments along with single AM fungal species and bacterial genotypes (Jiang et al., 2021; Faghihinia et al., 2022), but the expansion of these experimental designs to incorporate more diverse microbiota could improve biogeochemical processing within the AM fungal-bacterial relationship. These interactions between bacteria and AM fungi indicate the distinct physiological and ecological advantages that AM fungi contribute to the rhizosphere microbiome and increased accessibility of soil bacteria to nutritional pockets ideal for biogeochemical cycling.

### 2.4 The relevance of AM fungal functional traits for understanding microbial interaction dynamics

The complex relationships between AM fungal morphology, taxonomy, and functional traits suggests that phylogenetic classification of AM fungi based on functional traits is far from being established (Sbrana et al., 2014; Zanne et al., 2020). Although more research has investigated ectomycorrhizal fungal functional evolution (Miyauchi et al., 2020), it is still unclear how AM fungal families differ in function and how this is related to taxonomy. Attempts to elucidate these patterns (Chagnon et al., 2013) have suggested that the taxonomic composition of AM fungal communities cannot solely be used to predict AM fungal functionality. As Munkvold et al. found in (2004), AM fungal communities with low species diversity may still have considerable heterogeneity in their functional representation and contributions to the rhizosphere. However, we have yet to adequately quantify AM fungal functional traits and their influence on the sustainability of soil systems (Chaudhary et al., 2020). To gain more relevance to soil systems, researchers should evaluate interaction of soil bacteria with AM fungi based on different AM fungal families, or genotypes, to identify how taxonomy influences bacterial biogeochemical functioning.

While the direct effects of AM fungi’s contributions to rhizosphere microbiome function are currently still under investigation, it is likely that AM fungi’s presence and role as a foundational group in the rhizosphere has indirectly increased the *functional diversity* (*see **Box 1** for glossary of terms*) and resiliency of their associated plants. AM fungal communities have been shown to contribute to increased higher rhizosphere microbiome functional diversity when groups of functionally diverse AM fungal taxa are present (Ceccarelli et al., 2010; Yuan et al., 2019). Functionally diverse AM fungal communities have been shown to enhance host plant secondary metabolite production and *metabolic plasticity (see **Box 1**.)*, which increases plant adaptations to environmental stressors (Albrechtova et al., 2012; Hart et al., 2015; Avio et al., 2018; Rivero et al., 2018). These secondary metabolites are thought to play a fundamental role in recruitment of plant health-promoting bacteria and increasing functional diversity of the rhizosphere microbiome (Agnolucci et al., 2015; Turrini et al., 2018; Agnolucci et al., 2020).

Despite the great potential that understanding AM fungal-bacterial relationships presents for improving agricultural and other terrestrial ecosystems, a major knowledge gap exists in understanding how AM fungal functional traits contribute to microbiome dynamics and ecosystem processes. Furthermore, compared to the vast research in AM fungal ecology, few studies have considered incorporating bacterial communities, thereby excluding the important role that rare bacterial taxa play in enzyme activity and the selective pressures that lead to the coevolution of microbiome function (Guttman et al., 2014; Xiong et al., 2020). Experimental manipulations of soil microbial communities can lead to a better understanding of AM fungal-bacterial relationships and their influence on host plant secondary metabolic exudation. For example, mock, or synthetic, bacterial communities could be constructed and established in the rhizosphere with and without AM fungi. It may then be possible to determine how the presence of AM fungi influences plant secondary metabolic production and if these metabolites change bacterial community structure. By studying how AM fungi influence plant secondary metabolic production and indirectly influence bacterial communities, we may begin to understand AM fungi functional traits more clearly. 

## 3 Methods to determine AM fungal functional traits and applications

### 3.1 Utilizing synergistic properties and predictive tools to build hypotheses about AM fungal functional traits

Microbe-microbe interplay consists of important selective forces resulting in complex microbial assemblages that impact resource acquisition for host plants (Hassani et al., 2018). Much of the research investigating microbial interplay has been the result of careful experimental design with synthetic (or mock) communities (Liu et al., 2019). Much of this research on synthetic communities has ignored AM fungi, which is unfortunate given its keystone and near ubiquitous role in the rhizosphere (Jeffries et al., 2003; van der Heijden et al., 2015; Yuan et al., 2021). Recent studies have analyzed the metabolic facilitation of AM fungi and bacterial interactions in acquiring nutrients for host plants (Jansa and Hodge, 2021; Jiang et al., 2021; Nacoon et al., 2021). Much more research on AM fungal and bacterial synthetic microbial communities is needed to understand when AM fungal-bacterial relationships are complimentary or antagonistic. To understand the mechanisms by which AM fungi and bacteria facilitate each other, we can design experiments to assess how AM fungal communities and functional traits differ in the presence of bacteria from different phyla.

Subsequent research may include network analyses, which are often evaluated as co-occurrence networks where multiple correlations and models that can identify important taxa in facilitative microbial relationships and microbiome stability. In 2018, de Vries et al. found that soil bacterial co-occurrence networks were destabilized by drought in grassland systems, whereas fungal networks were more stable. Along with networks, de Vries et al. (2018) found that shifts in bacterial communities had greater effects on ecosystem functioning than fungi. Here, co-occurrence networks were used in conjunction with other analyses to understand the stability of microbial communities under stress as well their recoveries. Furthermore, networks can be used to understand important linkages between taxa. Scientists have found that fungal-bacterial networks provide insight into cooperative and competitive interactions (Zheng et al., 2018). Therefore, the utilization of network analyses can elucidate the types of interactions that occur between soil microorganisms, and under which circumstances they shift. The culmination of multiple network analyses may lead to changes in the way that we think about and evaluate relationships between soil organisms and the spatial scale at which they operate. Currently, the most efficient way to study these interactions is by observing patterns in microbial networks and using inferred data to dictate the questions and experiments that we design.

Nevertheless, there are certain limitations in the use of network analyses and their associated models, particularly with experimental design. To maximize the robustness of inferred networks, studies need to use a large number of replicates and should aim to have a large collection of samples and shared datasets to improve the predictive power of these models (Barroso-Bergadá et al., 2021). Network analyses have also received scrutiny due to their limited scope of inference based on mathematical projections, as opposed to quantifying the physical interactions between microbes (Röttjers and Faust, 2018; Hirano and Takemoto, 2019; Matchado et al., 2021). Since we lack empirical data pertaining to AM fungal-bacterial interactions and consistency within protocols that can differentiate these relationships from confounding environmental samples, we can only build knowledge from a portion of network analyses through careful interpretation and statistics (Guseva et al., 2022). Nonetheless, there is a vast variety of network analyses, and associated data (ie. closeness centrality, modularity) that could expand our current understanding of microbial communities within their surrounding environment and the predictive capability of these relationships, as opposed to analyzing taxa as isolated units. Pattern-based approaches can be used with large datasets in conjunction with machine learning algorithms, which have proven to be more powerful in creating predictive models compared to network analyses correlative power alone (Ramirez et al., 2018). The predictive power of machine learning can be used to identify potential ‘indicator’ taxa with more robust potential than core microbiome and hub taxa analyses by incorporating larger, more complex datasets that can factor biological attributes and functional complementarity within their algorithms (Shade and Handelsman, 2012; Qu et al., 2019; Thompson et al., 2019).

The attractiveness of pattern-based approaches such as network analysis is their applicability to environmental samples which are more easily available than experimental ones. To facilitate the use of pattern-based approaches, further steps can help to overcome their limitations. One step could be comparing networks across environmental gradients, chronosequences and timeseries. Comparisons of AM fungal-bacterial networks across spatial and temporal scales will enable to disentangle potential interactions from co-variation with environmental conditions and give insight into causality of interactions between AM fungal and bacterial communities (as e.g., in prey-predator dynamics) (Herold et al., 2020). Multi-omics approaches to extract community information such as metagenomics and metatranscriptomics represent another step and technique to enhance insights into species interactions from AM fungal-bacterial networks. Especially RNA-based approaches, contrary to DNA-based approaches, extract information on co-occurrences of shifts in living, active community structures (e.g., metatranscriptomics) which can be more directly linked to outcomes of species interactions (Hirano and Takemoto, 2019). Beyond focusing on taxonomic information of active parts of communities, multi-omics approaches also allow more functional insights (Gonzalez et al., 2018; Bharti et al., 2021). For instance, extracting functional gene abundances, which when implemented into network analysis could aid mapping the functional consequences of AM fungal-bacterial interactions, i.e., their impact on ecosystem processes. Knowledge of functional consequences additionally will help to elucidate the mechanisms behind cross-kingdom interactions such as AM fungi and bacteria. For instance, let us imagine finding co-occurrence of high abundances of phosphorus acquiring genes in AM fungi with high abundances of genes in bacteria responsible for nitrogen fixation and exchange with plants. This could indicate that host plants meet high phosphorus demands of N-fixation with the help of AM fungi (Neuenkamp et al., 2018). The last step to overcome limitations of pattern-based approaches is complementing co-occurrence networks with tools focusing more on species interactions; this could offer a way forward to overcome the difficulty of interpreting species interactions from co-occurrence networks (Cazelles et al., 2016). Examples of such tools are Markov networks (Harris, 2016) or dynamic (time-series based) methods such as convergent cross-mapping (Sugihara et al., 2012) or sparse S-mapping (Suzuki et al., 2017).

## 4 Implications of AM fungal-bacterial interactions for applied sciences

There is a pressing call for an increase in sustainable use of fertilization within agriculture and the application of AM fungi has gained attention as a means for more sustainable agriculture. Although AM fungal-bacterial interactions are the steppingstones to understanding nutrient support for plants, these cross-kingdom interactions have largely been ignored in agricultural treatments. Their role in supporting plant nutrition, root system development, and soil organic matter development hold great potential for crop production and maintaining sustainability of soil systems (Chen et al., 2019; Steffan et al., 2020). At the moment, the application of AM fungi is labor-intensive and mostly reserved for use with high-value crops because its effectiveness is largely dependent on a variety of abiotic factors at each site (Zheng et al., 2018). Large nutrient pools and microbial communities are often overlooked as agricultural conditions of concern but are key to increasing the efficiency of the soil system (Sosa-Hernández et al., 2019). Furthermore, AM fungal taxa vary in their associations with different bacterial genera (Yuan et al., 2021), indicating that specific AM fungal species can be used to attract specific bacteria for a particular function (Luthfiana et al., 2021).

Knowledge is growing on the potential use of AM fungal-bacterial relationships in agriculture, with promising results for future application in sustainable agricultural production. Various co-inoculation experiments have successfully identified relationships between AM fungal and rhizosphere microorganisms that help cultivate soil functions to improve agriculture (Barea, 2015; Mickan et al., 2019; Jiang et al., 2021). The field of rhizospheric microbiome engineering has seen an increase in recognition through the development of research on niche specificity and interactions in microbial communities (Naylor and Coleman-Derr, 2018). Rhizospheric microbiome engineering integrates knowledge from microbial communities and rhizosphere heritability to increase microbial efficiency through engineering (Kumawat et al., 2022). While field developments have been known to be inconsistent across many biofertilizers, understanding the mechanisms behind microbial interactions consisting of various members of the microbiome can help identify efficient candidates for use as biofertilizers (Pirttilä et al., 2021). Separating the effects of different microbial groups continues to be a challenge in studying AM fungi and bacteria, due to changes in shifting microbial proportions that make it difficult to understand if AM fungal functional traits are being outweighed by the functional traits of other microbial groups (Anthony et al., 2020). Nonetheless, identifying the effects of particular AM fungal traits is likely to lead to empirical data that can account for the AM fungal role in AM fungal-bacterial relationships.

## 5 Conclusions and future directions

By reviewing the current literature on AM fungal-bacterial relationships, we have gathered that these relationships are dependent on the community-oriented nature of soil microbiomes, by which microbial relationships are at the foundation of understanding microbiome functionality and deciphering microbial functional traits. For AM fungal functional traits, this seems to be dependent on their relationship with bacteria and the fluctuations of the bacterial communities present, which engage particular AM fungal taxa. We propose that AM fungal ecologists utilize both bacterial and AM fungal datasets to gain insights into AM fungal functional traits and to produce new knowledge pertaining to AM fungal-bacterial relationships. By collecting multi-omics datasets pertaining to AM fungal-bacterial relationships, along with sampling within and outside the rhizosphere (**Figure 1**), we can build new hypotheses that challenge: (1) if AM functional traits are conserved phylogenetically (2) how different AM fungi allocate carbon to microbes outside of the rhizosphere (3) And how bacterial communities influence AM fungal functional traits. We can use network tools such as hub formation to identify taxa that are highly connected within the network and core microbiome tools to identify if those hubs are core taxa (Agler et al., 2016). The inclusion of extended network tools focusing on species interactions (e.g. Markov networks or S-mapping) could further help identifying interactions from co-occurrences (Hirano and Takemoto, 2019). By incorporating bacterial communities, AM fungal ecologists can build insights into these dynamic interkingdom relationships that improve our understanding of AM fungal functional traits.

Prior to field applications of AM fungi in agriculture and other terrestrial ecosystems, it is fundamental that we understand how changes in AM fungal taxa will influence bacterial communities, along with microbiome and ecosystem functioning. It is important for AM fungal ecologists to utilize advanced computational tools as a predictive measure to distinguish which traits are dependent on bacterial community composition and which are inherent to AM fungal taxa. Studies that include pattern-based analyses, like network analyses, could help shed light on AM fungal-bacterial relationships and can be used to experimentally to tease apart interdependent microbial functions. Spatial and temporal replication of samples to assess relationship patterns will hereby enhance our understanding of the environmental impact on AM fungal bacterial relationships (Herold et al., 2020). Regarding experimental approaches, by manipulating particular key taxa that contribute to holobiont function, like AM fungi, through the use of synthetic or mock communities (Egan et al., 2018), we can build knowledge pertaining to specific AM fungal families or taxa and how they differ in interacting with microbial groups using controlled environment experiments. The use of synthetic communities has a great advantage over exclusionary treatments, like fungicide because of confounding chemical effects to soil chemistry. By utilizing technologies such as synthetic communities, and bringing tools together from different disciplines, we can overcome many of the obstacles pertaining to the identification of AM fungal functional traits.

Based on this review, we propose that understanding AM fungal-bacterial interactions are important for sustainable management of soil systems and that the identification of AM fungal functional traits can be attained through the analysis of AM fungal-bacterial relationships. Our literature synthesis draws inference to the interdependent nature of AM fungal-bacterial relationships and has suggested some of the data and tools that can be used to provide insights to AM fungal functional traits. In summary, we hope that the field of AM fungal ecology shifts its focus to identifying AM fungal functional traits, through the lens of AM fungal-bacterial relationships, to decipher the differences between their functional roles in the soil microbiome. In addressing this knowledge gap, we can use contextual evidence to infer and subsequently test which AM fungal functional traits are dependent on bacterial symbionts and which are phylogenetically conserved, improving microbial inoculum and products. Using these molecular advances from other interdisciplinary fields, new inferences and hypotheses could be made that inspire insightful methods moving forward.

## Author contributions

SH and LN conceptualized and designed the format of the manuscript. SH wrote the manuscript and created the figure and box. LN critically reviewed and revised the manuscript. LN and MP edited the figure. SH, LN, PT, and MP contributed to the literature presented in the review. PT and MP edited the manuscript and supervised the paper. All authors contributed to the article and approved of the submitted version.
